# Simulation of Left Atrial Function Using a Multi-Scale Model of the Cardiovascular System

**DOI:** 10.1371/journal.pone.0065146

**Published:** 2013-06-03

**Authors:** Antoine Pironet, Pierre C. Dauby, Sabine Paeme, Sarah Kosta, J. Geoffrey Chase, Thomas Desaive

**Affiliations:** 1 University of Liège (ULg), GIGA-Cardiovascular Sciences, Liège, Belgium; 2 Department of Mechanical Engineering, University of Canterbury, Christchurch, New Zealand; University of Adelaide, Australia

## Abstract

During a full cardiac cycle, the left atrium successively behaves as a reservoir, a conduit and a pump. This complex behavior makes it unrealistic to apply the time-varying elastance theory to characterize the left atrium, first, because this theory has known limitations, and second, because it is still uncertain whether the load independence hypothesis holds. In this study, we aim to bypass this uncertainty by relying on another kind of mathematical model of the cardiac chambers. In the present work, we describe both the left atrium and the left ventricle with a multi-scale model. The multi-scale property of this model comes from the fact that pressure inside a cardiac chamber is derived from a model of the sarcomere behavior. Macroscopic model parameters are identified from reference dog hemodynamic data. The multi-scale model of the cardiovascular system including the left atrium is then simulated to show that the physiological roles of the left atrium are correctly reproduced. This include a biphasic pressure wave and an eight-shaped pressure-volume loop. We also test the validity of our model in non basal conditions by reproducing a preload reduction experiment by inferior vena cava occlusion with the model. We compute the variation of eight indices before and after this experiment and obtain the same variation as experimentally observed for seven out of the eight indices. In summary, the multi-scale mathematical model presented in this work is able to correctly account for the three roles of the left atrium and also exhibits a realistic left atrial pressure-volume loop. Furthermore, the model has been previously presented and validated for the left ventricle. This makes it a proper alternative to the time-varying elastance theory if the focus is set on precisely representing the left atrial and left ventricular behaviors.

## Introduction

### Left Atrial Function

The left ventricle (LV) functions as a pump whose role is to drive blood flow through the vessels to the systemic circulation. For this reason, most researchers concentrated their efforts on trying to understand the ejection capabilities of the heart during systole, according little attention to the second heart chamber located upstream, namely, the left atrium (LA). More recently, the study of the heart's diastolic behavior, the way in which the heart fills, has gained interest since impaired filling implies impaired ejection. This direction naturally led researchers to focus on the LA and on the way it modulates left ventricular filling. In particular, they found that left atrial contraction can account for between 15 and 30% [1]–[3] of left ventricular filling. Consequently, there is interest in understanding how this second heart chamber behaves. Study of the left atrial behavior can be performed using a variety of measuring techniques, such as echography [1]–[4], angiography [2], computed tomography [2], [4], magnetic resonance imaging [2]–[4] and invasive catheterization [2], [3].

**Figure 1:**
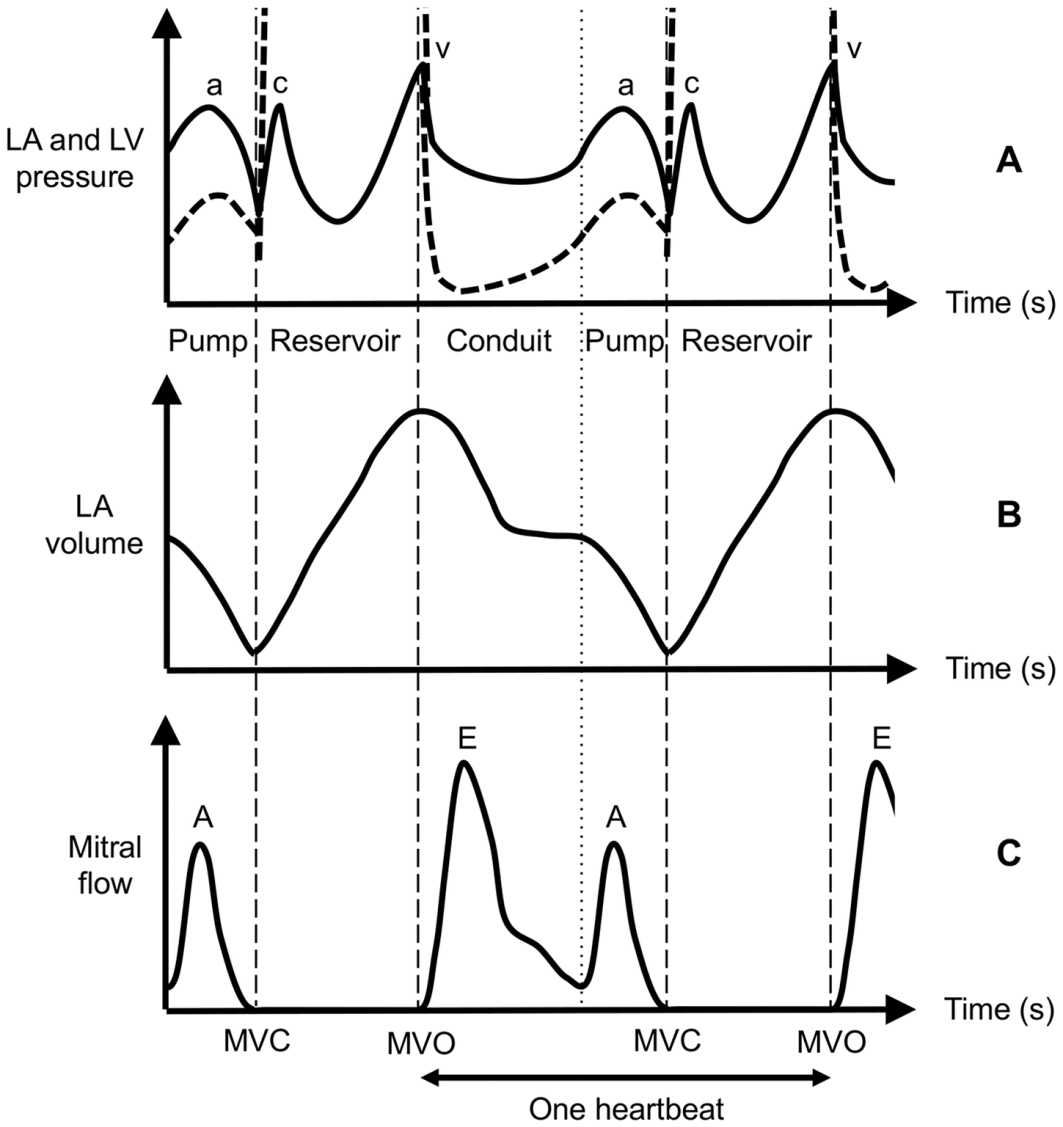
Evolution of atrial and ventricular variables during a cardiac cycle. *Panel A*: left ventricular (dashed line) and left atrial (full line) pressures with specific waves (a, c and v waves) indicated. *Panel B*: left atrial pressure. *Panel C*: transmitral flow with specific waves (E and A waves) indicated. Opening (MVO) and closing (MVC) times of the mitral valve are also indicated.

During a cardiac cycle, the left atrium exerts three different roles [1], [2]. First, it provides a reservoir for blood during ventricular contraction when mitral valve is closed. After the mitral valve opens, the LA behaves as a conduit to passively fill the left ventricle during the initial filling period of the cardiac cycle. During the last part of filling, the atrium actively contracts as a pump to further fill and pressurize the LV. The reservoir and pump phases lead to what are referred to as the “v” and “a” wave portions of the atrial pressure curve. The typical evolution of LA and LV pressures and LA volume is shown in Figure 1 (panels A and B). As a consequence of left atrial behavior, flow through the mitral valve during a cardiac cycle also exhibits a biphasic behavior, as shown in Figure 1 (panel C). When mitral valve is completely closed, transmitral flow is obviously zero. During the first, passive phase of ventricular filling, transmitral flow exhibits a peak, termed the “E wave”. Then, when the ventricle is relaxed and before the atrium contracts, the transmitral flow is nearly zero. This phase is called “diastasis” [5]. Finally, when the left atrium actively contracts, transmitral flow peaks again. This second peak is called the “A wave”.

**Figure 2:**
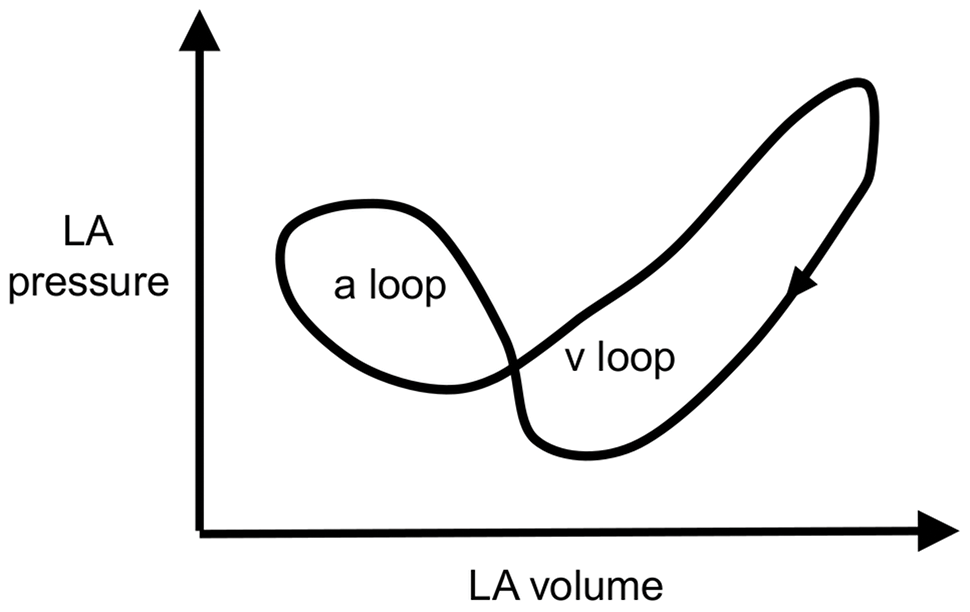
Sketch of a typical left atrial pressure-volume loop. This loop consists of two lobes: the a loop and the v loop.

When atrial pressure and volume are plotted against one another during a full cardiac cycle, the result is a closed curve in the pressure-volume plane, called the atrial pressure-volume (PV) loop. An example of such a loop is given in Figure 2. This loop is composed of two lobes, giving it a particular figure-eight shape. The right lobe (higher volumes) is the “v loop” and represents the passive properties of the atrium, namely the reservoir and conduit functions. The left lobe (lower volumes) is the “a loop”, which is caused by active contraction of the atrium.

As far as the LV is concerned, Suga *et al.* [6] plotted experimental PV loops for various loading conditions exerted on the LV. Linear regression to determine an elastance (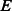) at every point in the cardiac cycle yielded a time-varying elastance 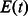. The main findings using this method were that 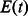 was independent of the load exerted on the ventricle and cardiac period, and that dead space volume (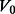) was not variable. This finding implies that, using this function 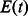, ventricular pressure can be computed from ventricular volume and *vice versa*, and this computation can be done for any loading condition of the ventricle. Thus, the concept of time-varying elastance is a powerful concept widely used by clinicians to account for the heart's behavior.

Since the work of Suga *et al.*, many authors expressed doubt about the characterization of the LV by a time-varying elastance function. First, many authors did not prefer the lack of physiological grounds of this empirical theory and argued that modelling the ventricular behavior should rely on a model of the sarcomere [7]. Second, some researchers experimentally observed a parabolic (rather than linear) relation between instantaneous ventricular pressure and volume [8], [9]. The time-varying elastance theory could be modified to account for these experimental findings. Third, other experiments showed the dependence of ventricular pressure on flow [10], [11], which led to another modification of the time-varying elastance theory to include this “resistive” effect. Finally, some authors found a dependence of 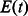 on load [12] and subject condition [13], [14], thus violating the fundamental hypothesis of the time-varying elastance theory of Suga *et al.*, namely the uniqueness of the 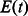 curve. These four points are detailed in a recent article [15], in which we developed a multi-scale model of the cardiovascular system that is able to account for all these drawbacks of the time-varying elastance theory for the LV.

The present study seeks to extend the previous multi-scale model to the LA to gain a more complete understanding. Indeed, the application of the time-varying elastance concept to the LA is much more uncertain. To our knowledge, only two groups have computed an experimental time-varying elastance curve for the LA [16], [17], of which only the first tested the load independence of the atrial 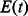 and came up with a negative conclusion. More recently, other groups [18]–[20] also plotted pressure-volume loops for differently loaded left atrial beats, bud did not explicitly compute an atrial elastance curve.

Thus, the load independence of the atrial 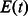 in general remains an open question. What prior studies agree on is that the atrial 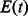 curve is bimodal. However, probably for simplifying purposes, atrial models available in the literature only use unimodal elastance curves [21]–[28]. Thus, a multi-scale model analysis can provide clarity on these issues. This work thus relies on a characterization of both left atrial and left ventricular contractility based on a multi-scale model. The resulting model will be connected to a validated model of the right ventricle and circulation that uses a time-varying elastance for the right ventricle and used to test these questions *in silico*. The model will be validated by comparison with experimental data collected on dogs [8], [20], [29].

## Methods

### Multi-scale Cardiovascular System Model

**Figure 3:**
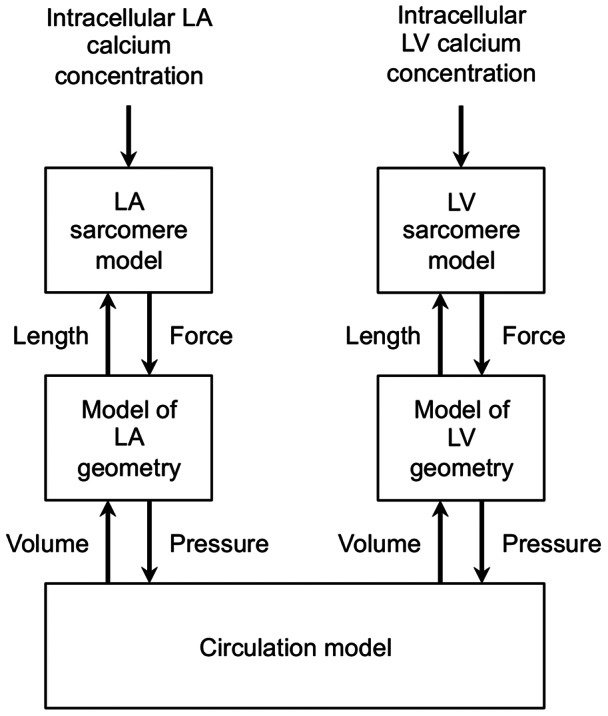
Interconnection of the models with inputs and outputs of each model.

The model we develop and use in this work is an assembling of existing models on different scales. Figure 3 summarizes these models and their connection to the circulation. The model inputs consist of two curves describing the time course of intracellular calcium concentration in atrial and ventricular cells. Each of these inputs is fed into a model of the sarcomere behavior, which describes the way sarcomeres generate force from their contraction and the concentration of cross-bridges. One sarcomere model represents the atrial sarcomere, and the other the ventricular sarcomere. The sarcomere models are in fact the same, but the values of some model parameters are different for the ventricle and the atrium. Force generated by the sarcomeres is then converted to pressure using Laplace's law and simple geometrical models of the LA and LV are built, which allows to link chamber volume to sarcomere length.

#### Calcium inputs

Reference curves depicting the time course of intracellular calcium concentration in the left ventricle and left atrium were used as model inputs. The reference curve for the left ventricle is taken from an experimental study on ferrets [30], while the reference curve for the left atrium is extracted from simulations of mathematical model of a human atrial cell [31]. These curves were both fitted with cosine functions, yielding smooth input curves for the numerical model, and stretched to account for a heart period of 0.45 s [20]. This approach gave the following equation for intracellular calcium concentration in the LV and LA:
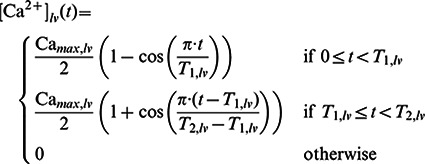
(1)

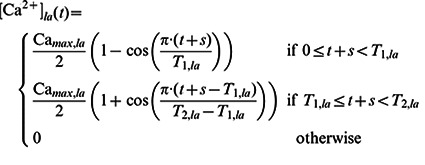
(2)


#### Left atrial and left ventricular sarcomere model

The heart sarcomere model used in this work has been presented by Negroni and Lascano [32]. Equations describing the equivalent sarcomere behavior from a chemical and mechanical point of view have also been presented before [15], [32], [33] and are just recalled here:
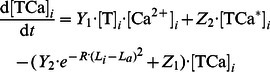
(3)

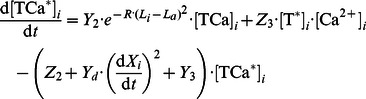
(4)

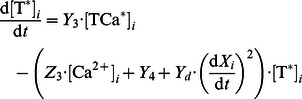
(5)

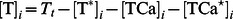
(6)

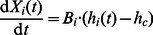
(7)

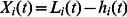
(8)

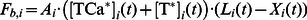
(9)

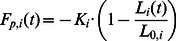
(10)

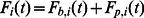
(11)


In all the previous equations, the subscript 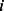 denotes either the LV or the LA. The meaning and units of all model variables are presented in Table 1. As a difference with our previous work, passive force generated by a chamber 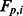 is computed with a linear relation (Equation 10), instead of a fifth order relation. This assumption has been made by others [34] and has the advantage of simplifying the parameter identification process (see Text S1). The series elastic element introduced by Negroni and Lascano in a more complex version of their model [33] has also been omitted here.

**Table 1:** Model variables.

Symbol	Variable	Units
[Ca^2+^]*_i_*	Concentration of intracellular calcium	*μ*M
[TCa]*_i_*	Concentration of troponin C bound with calcium (cross-bridges attached)	*μ*M
[TCa^*^]*_i_*	Concentration of troponin C bound with calcium (cross-bridges detached)	*μ*M
[T^*^]*_i_*	Concentration of troponin C not bound with calcium (cross-bridges attached)	*μ*M
[T]*_i_*	Concentration of troponin C not bound with calcium (cross-bridges detached)	*μ*M
*L_i_*	Length of the equivalent half sarcomere	*μ*m
*h_i_*	Length of the equivalent cross-bridge	*μ*m
*X_i_*	Length of the equivalent half sarcomere minus length of the cross-bridge	*μ*m
*F_b,i_*	Passive force generated by the equivalent half sarcomere	mN/mm^2^
*F_a,i_*	Active force generated by the equivalent half sarcomere	mN/mm^2^
*F_i_*	Total force generated by the equivalent half sarcomere	mN/mm^2^

#### Cardiac chamber model

Another difference from our previous work is the model used to represent a cardiac chamber. In this work, we choose to use the model developed by Shim *et al.* [34], where cardiac chambers are supposed to be hemispheric. Pressure 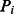 in a cardiac chamber is computed using Laplace’s law:



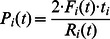
(12)where 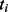 is the thickness of the chamber and 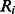 is its radius, computed from the chamber volume using the following equation:



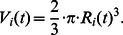
(13)The link from micro to macroscopic scales is made by assuming that radial deformation of the ventricle is equal to deformation of the sarcomere:
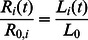
(14)where 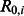 is the unstressed chamber radius and 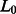, the unstressed sarcomere length.

#### Cardiovascular system model

To completely describe the cardiovascular system (CVS), models of the left atrial and left ventricular chambers that have been presented in the previous section were coupled to lumped models of other important parts of the cardiovascular system, namely: the aorta, the vena cava, the right ventricle, the pulmonary artery and the pulmonary veins. These were represented as chambers, linked by flow resistances [35]. Elements that are described in this section (passive chambers, time-varying elastance chambers, resistances and valves) are taken from a previously published model of the CVS [35]. This model has been validated both *in silico* [36] and in animal experiments [37]–[39].

First, the aorta (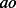), vena cava (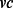), pulmonary artery (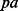) and veins (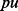) were represented as passive chambers, whose pressures 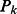 and volumes 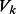 are linked by a constant, denoted 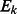 and called the elastance. Hence,
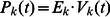
(15)where 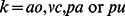.

Since the focus of this article is set on the left atrium, we choose to neglect the right atrium. Its function is merged in that of the right ventricle (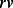), which is described using the widespread time-varying elastance concept [24], [35]. This means that right ventricular pressure 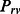 and volume 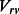 are also linked by a (not constant) elastance parameter, namely:
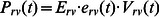
(16)where 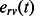 is denoted the time-varying elastance of the right ventricle and 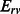 is a normalization constant. The time-varying elastance function is usually decomposed into Gaussian functions [24], as:



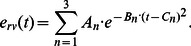
(17)Values of the constants 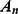, 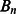 and 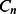 have been taken from [24] and are given in Table 2.

**Table 2:** Values of the model parameters.

Parameter	Value	Units	Source
Intracellular calcium concentrations			
*T*_1,*lv*_	0.0305	S	Computed from Yue *et al.* [30]
*T*_2,*lv*_	0.0977	S	Computed from Yue *et al.* [30]
*Ca_max,lv_*	1.47	mM	Computed from Yue *et al.* [30]
*T*_1,*la*_	0.0084	S	Computed from Nygren *et al.* [31]
*T*_2,*la*_	0.0300	S	Computed from Nygren *et al.* [31]
*Ca_max,la_*	1.33	mM	Computed from Nygren *et al.* [31]
*s*	0.085	s	Computed from Gare *et al.* [20]
Chemical kinetics			
*Y*_1_	39	mM⋅s^–1^	Negroni and Lascano [33]
*Z*_1_	30	s^–1^	Negroni and Lascano [33]
*Y*_2_	1.3	s^–1^	Negroni and Lascano [33]
*Z*_2_	1.3	s^–1^	Negroni and Lascano [33]
*Y*_3_	30	s^–1^	Negroni and Lascano [33]
*Z*_3_	1560	mM⋅s^–1^	Negroni and Lascano [33]
*Y*_4_	40	s^–1^	Negroni and Lascano [33]
*Y_d_*	9	s⋅mM^–2^	Negroni and Lascano [33]
*T_t_*	70	mM	Negroni and Lascano [33]
*B*	1200	s^–1^	Negroni and Lascano [33]
*h_c_*	0.005	mm	Negroni and Lascano [33]
*L_a_*	1.17	mm	Negroni and Lascano [33]
*R*	20	mm^–2^	Negroni and Lascano [33]
Cross-bridge parallel and elastic forces			
*A_la_*	577.51	mN⋅mm^–2^⋅mm^–1^⋅mM^–1^	Adjusted from Gare *et al.* [20]
*K_la_*	20.00	mN⋅mm^–2^⋅mm^–5^	Adjusted from Gare *et al.* [20]
*A_la_*	944.58	mN⋅mm^–2^⋅mm^–1^⋅mN^–1^	Adjusted from Gare *et al.* [20]
*K_lv_*	0.4853	mN⋅mm^–2^⋅mm^–5^	Adjusted from Gare *et al.* [20]
*L*_0_	0.97	mM	Negroni and Lascano [33]
Force-length to pressure-volume conversion			
*R*_0,*la*_	1.66	cm	Computed from Gare *et al.* [20]
*t_la_*	1.98	cm	Adjusted from Gare *et al.* [20]
*R*_0,*lv*_	1.62	cm	Computed from Kass *et al.* [8]
*t_lv_*	6.36	cm	Adjusted from Kass *et al.* [8]
Hemodynamic parameters			
*E_pa_*	2.29	mmHg ml^–1^	Computed from Maughan *et al.* [29]
*E_pu_*	0.0881	mmHg ml^–1^	Adjusted from Gare *et al.* [20], Kass *et al.* [8] and Maughan *et al.* [29]
*E_ao_*	6.94	mmHg ml^–1^	Computed from Gare *et al.* [20], Kass *et al.* [8] and Maughan *et al.* [29]
*E_vc_*	1.3077	mmHg ml^–1^	Computed from Gare *et al.* [20], Kass *et al.* [8] and Maughan *et al.* [29]
*E_rv_*	2.10	mmHg ml^–1^	Adjusted from Maughan *et al.* [29]
*R_pul_*	2.454	mmHg s⋅ml^–1^	Adjusted from Maughan *et al.* [29]
*R_sys_*	3.61	mmHg s⋅ml^–1^	Computed from Gare *et al.* [20], Kass *et al.* [8] and Maughan *et al.* [29]
*R_av_*	0.0846	mmHg s⋅ml^–1^	Computed from Gare *et al.* [20] and Kass *et al.* [8]
*R_mt_*	0.0278	mmHg s⋅ml^–1^	Computed from Gare *et al.* [20] and Kass *et al.* [8]
*R_pv_*	0.03	mmHg s⋅ml^–1^	Revie *et al.* [49]
*R_tc_*	0.279	mmHg s⋅ml^–1^	Adjusted from Gare *et al.* [20], Kass *et al.* [8] and Maughan *et al.* [29]
*R_prox_*	0.108	mmHg s⋅ml^–1^	Computed from Gare *et al.* [20] and Kass *et al.* [8]
Stressed blood volume	273	ml	Computed from Burkhoff and Tyberg [50] and Finsterer *et al.* [51]
Right ventricle driver function			
*A*_1_	0.955	–	Chung *et al.* [24]
*A*_2_	0.624	–	Chung *et al.* [24]
*A*_3_	0.018	–	Chung *et al.* [24]
*B*_1_	454	s^–2^	Adapted from Chung *et al.* [24]
*B*_2_	400	s^–2^	Adapted from Chung *et al.* [24]
*B*_3_	7511	s^–2^	Adapted from Chung *et al.* [24]
*C*_1_	0.1745	s	Adapted from Chung *et al.* [24]
*C*_2_	0.097	s	Adapted from Chung *et al.* [24]
*C*_3_	0.143	s	Adapted from Chung *et al.* [24]
Cardiac period	0.45	s	Computed from Gare *et al.* [24]

Flow resistances link the seven model chambers, accounting for the pressure drop between these chambers. The model comprises seven flow resistances, namely the systemic resistance (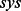), the pulmonary resistance (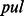), the mitral (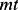), aortic (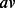), tricuspid (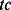) and pulmonary (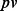) valves resistances and the resistance of the proximal section of the pulmonary vein (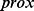). Flow 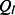 in a resistance between two chambers is then computed using Poiseuilles law for flow, namely:
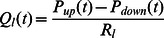
(18)where 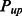 and 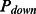 respectively represent pressures in chambers located up and downstream the resistance 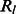, with 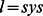, 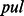, 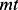, 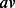, 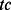, 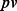 or 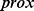.

**Figure 4:**
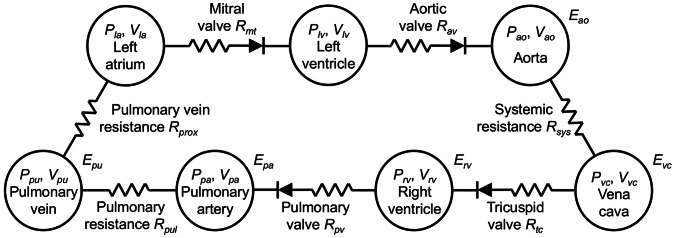
Cardiovascular system model. The seven model chambers are represented as circles, joined by flow resistances. Valves (modeled as diodes) are also found at the input and output of each ventricle.

In the heart, valves are located at the input and output of both ventricles, and close to prevent backward flow from the arteries to the ventricles or from the ventricles to the atria. In this work, valves are modeled as perfect electrical diodes, thus causing flow through a valve to be zero if the pressure gradient through that valve is negative. Consequently, for mitral, aortic, tricuspid and pulmonary valves, Equation 18 is modified as follows:



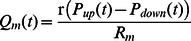
(19)where 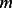 is either 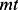, 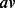, 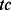 or 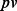 and 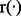 denotes the ramp function, defined as:



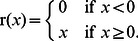
(20)Finally, volume of the chambers can be computed thanks to the continuity equation applied to all model chambers:
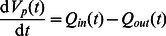
(21)where 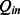 and 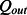 are respectively flows coming in and going out of the chamber. The subscript 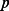 denotes all model chambers, namely 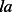, 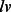, 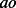, 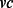, 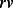, 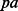 or 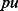. The complete cardiovascular system model is represented in Figure 4.

### Parameter Identification

In order to be able to simulate our model, we first had to assign values to the numerous parameters involved in the equations presented in the previous section. We chose to adjust the parameters of our model so that it correctly represented the hemodynamics of a normal dog, because reference data including left atrial pressure and volume was available in the literature for such animals [8], [20], [29]. Since the focus was set only on macroscopic hemodynamic values, we did not perform any adjustment on the micro-scale parameters of the sarcomere model. The detailed parameter adjustment process can be found in Text S1 and adjusted parameter values are shown in Table 2.

### Analyses

The identified model was first simulated to see if it properly accounted for the CVS behavior in general and for the left atrial behavior in particular. For this purpose, we qualitatively compared the behavior of the CVS model with reference curves for dogs published in the literature [8], [20], [29].

After checking that our atrial model behaves as physiologically expected, we tested the validity of our atrial model in non basal situations. To do so, we numerically reproduced preload reduction experiments carried out by Courtois *et al.* [40]. Preload denotes the initial stretching of the heart muscle before contraction [41]. In experimental settings, a way to achieve preload reductions is to occlude (with an inflatable balloon) the inferior vena cava, hence decreasing venous return to the left atrium. Courtois *et al.* [40] performed inferior vena cava occlusion (IVCO) experiments on closed-chest dogs and studied the variation of many indices just before and during 5 heartbeats following this maneuver. These indices were related to atrial and ventricular pressure and Doppler time-velocity profile through the mitral valve.

We reproduced IVCO experiments with our model by a fourfold increase of the pulmonary vascular resistance 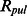. As experimentally done by Courtois *et al.*, we observed the variation of measurements before and 5 heartbeats after modification of 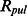. These measurements were: maximum a and v wave pressures, minimum and end-diastolic ventricular pressure, slopes of the a and v waves and maximum transmitral pressure gradients during early and late ventricular filling.

**Figure 5:**
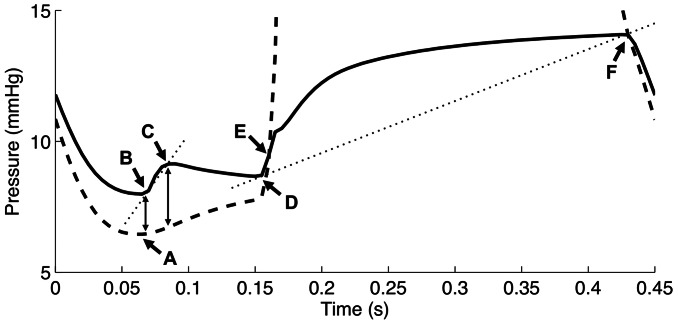
Indices used to assess the influence of IVCO experiments. Left atrial pressure is depicted in full line and left ventricular pressure is drawn in dashed line. The indices are: maximum a (*point C*) and v wave pressure (*point F*), minimum (*point A*) and end-diastolic (*point E*) ventricular pressures, slopes of the a (*dotted line BC*) and v (*dotted line DF*) waves and maximum transmitral pressure gradients during early (*left double arrow*) and late (*right double arrow*) ventricular filling.

The above mentioned measurements were computed by detecting 6 points on the left atrial and ventricular pressure curves (see Figure 5): (A) minimum left ventricular pressure, (B) onset of the a wave on left atrial pressure, (C) pressure of the a wave on left atrial pressure, (D) onset of the v wave on left atrial pressure, (E) crossover of the left ventricular and atrial pressures corresponding to closing of the mitral valve, (F) crossover of the left ventricular and atrial pressures corresponding to opening of the mitral valve. Maximum a and v wave pressures are, respectively, the ordinates of points C and F and minimum and end-diastolic ventricular pressures are, respectively, the ordinates of points A and E. Slopes of the a and v waves were computed as the slopes of the BC and DF lines, respectively. Finally, maximum transmitral pressure gradients before and after onset of atrial contraction were also computed. They are indicated by double arrows on Figure 5.

All computations, including parameter identification and model simulations were carried out using MATLAB (2011a, MathWorks, Natick, MA). The MATLAB code of our model is available as File S1.

## Results and Discussion

### Cardiovascular System Behavior

**Figure 6:**
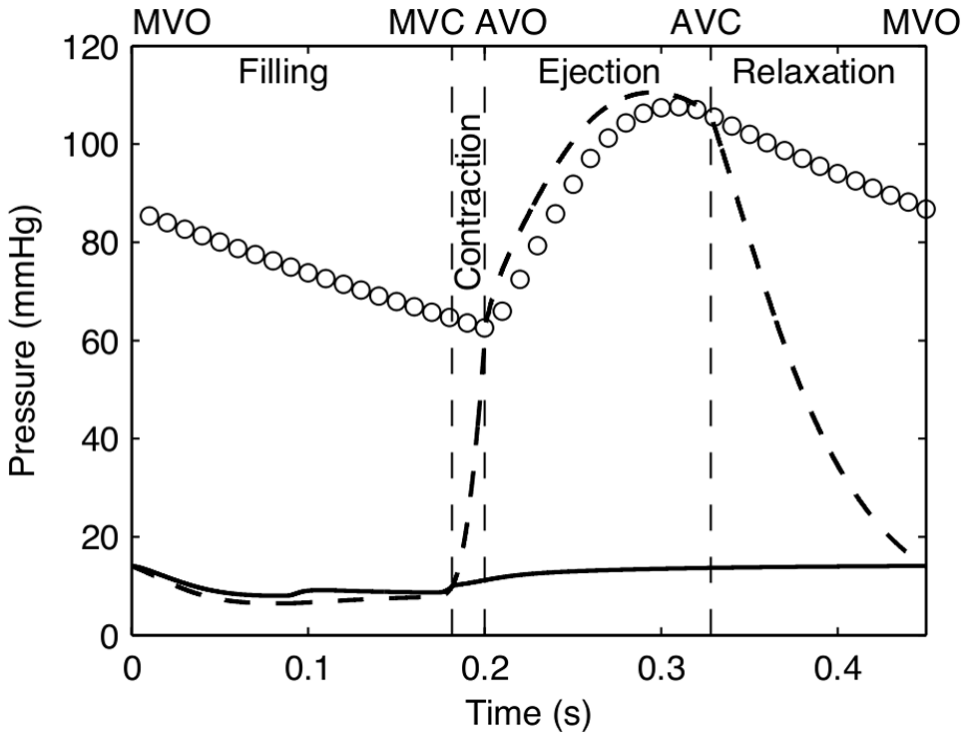
Evolution of simulated left atrial, left ventricular and aortic pressures during a cardiac cycle. *Solid line*: LA pressure, *dashed line*: LV pressure, *white dots*: aortic pressure. Opening and closing times of the mitral (MVO and MVC) and aortic valves (AVO and AVC) are also displayed. These events separate the four phases of the cardiac cycle: filling, contraction, ejection and relaxation.

Figure 6 displays simulated left atrial (solid line), left ventricular (dashed line) and aortic (white dots) pressures during a cardiac cycle. The four phases of ventricular behavior, namely filling, contraction, ejection and relaxation, are clearly distinguishable in the figure. The figure starts with opening of the mitral valve (MVO) and filling of the left ventricle. At the beginning of cardiac contraction, left ventricular pressure increases and causes closing of the mitral valve (MVC). The ventricle then contracts at a constant volume until ventricular pressure crosses over aortic pressure. At that moment, aortic valve opens (AVO) and the ventricle starts to eject. Aortic valve closes (AVC) when ventricular pressure drops under aortic pressure, which marks the beginning of the relaxation phase. This phase ends with opening of the mitral valve and the cycle goes on. The modeled cardiovascular behavior is thus physiologically realistic, compared to [42].

The same behavior can be qualitatively observed in the pulmonary circulation, in which pressures are lower. Systolic and diastolic pulmonary artery pressures equal 71.52 and 55.18 mmHg, which is consistent with reference right ventricular pressure [29], but which seems high compared to other published experimental dog data [43]. This issue comes from the fact that the right ventricular reference data we used has been derived from preload variation experiments. This is a drawback of using data sets coming from different experiments. Our model would benefit from a complete data set including left atrial pressure and volume coming from the same animal, but, to our knowledge, such data is not currently available in the literature. This inconsistency in the data does not compromise the overall functioning of the model and the study results.

### Atrial Behavior

**Figure 7:**
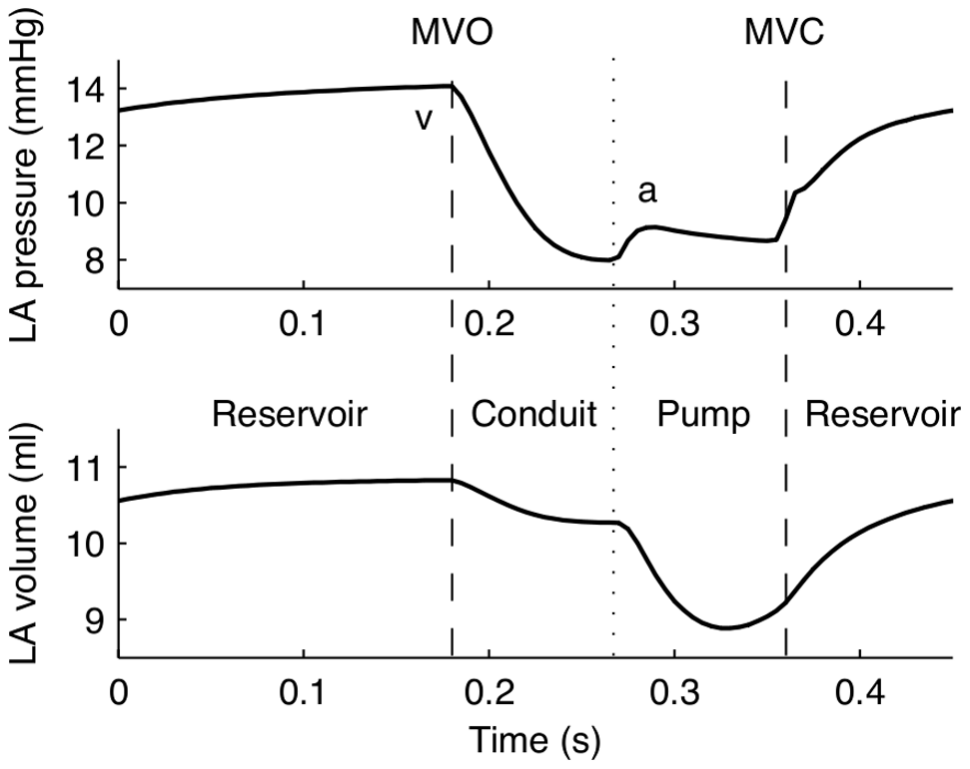
Simulated left atrial pressure and volume during one cardiac cycle.

Model simulations of the atrial behavior using adjusted parameter values were performed to check the validity of the model. For example, the time course of left atrial pressure and volume are shown in Figure 7. In this figure, the succession of events during one cardiac cycle is displayed. The figure begins during ventricular contraction, when mitral valve is closed. Consequently, blood accumulates in the atrium, hence increasing atrial volume and pressure. This is the reservoir function of the atrium. At the end of ventricular contraction, ventricular pressure decreases until it crosses atrial pressure. At this moment, mitral valve opens and blood starts flowing passively to the ventricle. This is the conduit function of the atrium. Atrial pressure thus reaches a maximum at the time of MVO. This maximum is the v wave and can be seen on model simulations. Finally, at the end of ventricular filling, the atrium contracts, increasing its pressure and decreasing its volume. The resulting peak in pressure is the a wave, which is correctly represented by the model. This is the pump function of the atrium. After atrial contraction, the mitral valve closes and the cycle goes on.

Comparison of Figures 1 and 7 shows that the model correctly accounts for the three roles of the atrium: reservoir, conduit and pump. The model is thus able to simulate the a and v waves, but the c wave is not represented. This is inherent to the lumped parameter nature of our model of the circulation. Indeed, the origin of the c wave is due to pressure wave propagation [2], that cannot occur in such models [22]. Consequently, an attempt to reproduce the c wave in our model would be purely artificial. Furthermore, the appearance of the c wave on left atrial pressure tracings is not systematic [1], [3].

**Figure 8:**
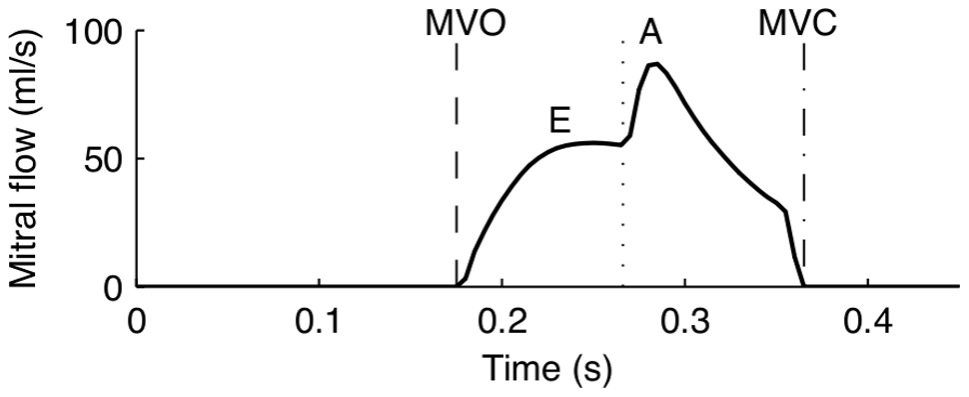
Simulated flow through the mitral valve during one cardiac cycle. Letters E and A denote the two characteristic waves of the mitral flow.

The time course of flow through the mitral valve resulting from model simulations is represented in Figure 8. As a consequence of the atrial behavior explained above, flow through the mitral valve exhibits two distinct components, namely the E and A waves, as can be seen in Figure 8. The E wave is the consequence of the passive ventricular filling while the atrium is in the conduit phase. When the atrium contracts, mitral flow increases once again; this increase is the A wave. The atrial contraction causes a supplementary increase of left ventricular volume. (Indeed, during filling, ventricular volume is the integral of mitral flow.) Model simulations are thus in good agreement with physiological reality.

Usually, E and A waves are separated by a phase during which mitral flow goes nearly back to zero, called diastasis, as shown in Figure 1. This phase is not present in the simulated mitral flow, because the cardiac period (0.45 s) computed from the reference data is quite small for a 25-kg dog [44], [45]. A small heart period implies a short diastole, as inspection of the reference data of Gare *et al.* [20] confirms. From a physiological point of view, a short diastole causes “fusion” of the E and A waves [46] and is accurate to our clinical observations. A longer observed period would lead to separated E and A waves with no change of method, showing the generality of the model and methods.

**Figure 9:**
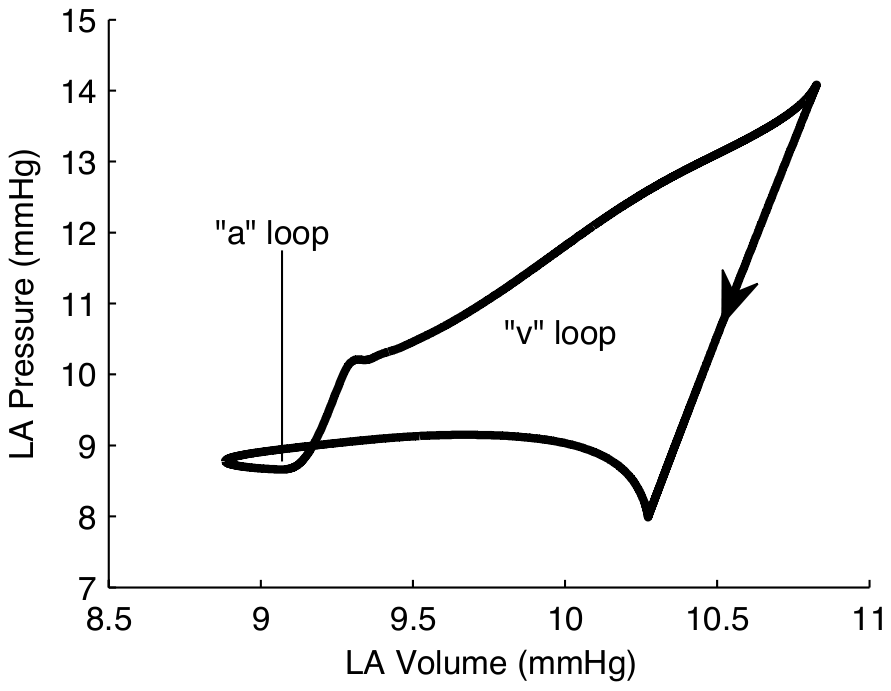
Simulated left atrial pressure-volume loop. This loop is composed of two distinct lobes: the “a” loop and the “v” loop.

Figure 9 depicts the left atrial pressure-volume loop. This simulated loop has the physiological figure-eight shape and consists of two lobes, one resulting from passive atrial properties and one from atrial contraction. This simulated loop thus correctly reflects the physiological behavior of the atrium. The simulated left atrial pressure-volume loop comprises a linear section at highest volumes, which is not observed in reality. This comes from the fact that a linear force-length relation was assumed for the passive force. The shape of this part of the loop could probably be improved by assuming a more detailed model for the passive force-length relation, instead of the linear relation given by equation 10.

One broader contextual limitation is that the model does not involve electrophysics. Hence, it cannot account for the interaction of left atrial function and electrophysiology, which remains an important challenge [47].

In summary, the model developed in this work is able to correctly reproduce the main features of the left atrial behavior. The overall accuracy of the atrial model is influenced by the rest of the CVS model elements, such as the valves, elastances and resistances. This work is only a first step in attempting to represent the LA, and the model would benefit from more precise models for these elements. This is particularly true for better valve models, especially for the mitral valve. Such models exist in the literature and works are in progress to investigate the potential benefits of using a more complex mitral valve model [48]. It is also worth noting that improved mitral valve models require improved LA models and *vice versa*. An incremental approach is thus necessary. On the other hand, the use of windkessel models to represent the arteries and veins is sufficient to meet or goals since the focus is set on the LA behavior.

### Simulation of Preload Reduction Experiments

The result of simulated preload reduction experiments through IVCO is presented in Table 3. This table shows the values of the atrial and ventricular indices before and after IVCO, for both the original experiments [40] and our model simulations. As can be seen from Table 3, all the 8 selected measurements followed the same decrease as experimentally observed.

**Table 3:** Comparison of experimental and simulated measurements before and after IVCO.

		Experiments [40]		Model simulations	
Measurement	Units	Baseline	IVCO	Baseline	IVCO
Maximum a wave pressure	mmHg	6.6	4.3	9.2	7.6
Maximum v wave pressure	mmHg	5.6	2.9	14	12
Minimum ventricular pressure	mmHg	1.0	–0.4	6.6	4.9
End-diastolic ventricular pressure	mmHg	6.5	3.8	9.7	8.5
Slope of the a wave	mmHg/s	60	37	53	38
Slope of the v wave	mmHg/s	21	13	20	16
Maximum early pressure gradient	mmHg	2.8	2.4	1.6	1.4
Maximum late pressure gradient	mmHg	1.2	0.9	2.4	2.2

Note also that we only included 8 measurements to assess the effects of IVCO, which is only the half of those used by Courtois *et al.* The reason is that, out of the 8 supplementary measurements, 4 were extracted from Doppler time-velocity profile through the mitral valve, which is not available from our model. The other 4 measurements could not be computed because they are based on features of the LV pressure waveform that are not reproduced by lumped parameter models, for example the reflection of the a wave on LV pressure.

Since the original experiments of Courtois *et al.* involved a modification of vena cava resistance, which is not explicitly represented in our model, we chose to model these experiments by a fourfold increase of the pulmonary vascular resistance 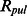.

Finally, it is necessary to emphasize that experimental and simulated values presented in Table 3 cannot be expected to match quantitatively, even for baseline situations, since model parameters were identified from other experimental data sets. Furthermore, even if the modeled and experimental baseline situations were identical, since the model presented here is quite simple, simulated variations would not necessarily be of the same order of magnitude as modeled variations, because of unmodeled features. The goal was only to check if the model quantitatively behaves as physiologically observed, which is clearly the case.

### Conclusions

In this article, we present a lumped, closed-loop model of the cardiovascular system taking the LA into account. Left atrial and left ventricular dynamics are described using a common model of sarcomere behavior. While the use of the time-varying elastance for description of the ventricular contraction is widely accepted, it is not the case for the LA, especially because is it is not sure whether the elastance curve is load-dependent or not. The alternative description we used is more complex than the purely empirical concept of time-varying elastance, but it is physiologically more relevant and allows to correctly and simultaneously describe the left ventricular and left atrial behaviors, while the former cannot.

Our model correctly accounts for the three main roles of the LA, and also exhibits a realistic pressure-volume loop. As a consequence of this proper representation of the atrial behavior, flow through the mitral valve also exhibits the physiologically observed features. Finally, we reproduced preload reduction experiments with the model, and showed that it followed the experimental reality. Consequently, this model provides a good alternative to the time-varying elastance theory if the focus is set on precisely representing the atrial behavior.

## Supporting Information

Text S1 **Parameter estimation process.**
(PDF)

File S1 **MATLAB code for model simulation.**
(M) Click here for additional data file.
