# Supplementary material for: Simulation of Left Atrial Function Using a Multi-Scale Model of the Cardiovascular System
Source: PLoS One. 2013 Jun 3;8(6):e65146. doi: 10.1371/journal.pone.0065146 (PMC3670859; doi:10.1371/journal.pone.0065146)
Supplement: Text S1 — Parameter estimation process. (PDF) [file pone.0065146.s001.pdf]

Supplementary Text S1: Parameter estimation

Step 1: direct parameter computation from data

Manipulating the CVS model equations and using reference data on dogs [1–3], we could directly compute the values of the hemodynamic parameters R_{prox}, R_{mt}, R_{av}, R_{sys}, E_{ao} and E_{pa}. The data published by Gare *et al.* [1] provide curves of left atrial pressure, left ventricular pressure and aortic pressure for one cardiac cycle. To compute the model parameters, we also needed the value of the stroke volume SV. It was computed from a canine pressure-volume loop published by Kass *et al.* [2] as:

$$SV \approx 11.67 \text{ ml}$$

Mean vena cava pressure $\bar{P}_{vc}$ was computed from a previously published right ventricular canine pressure-volume loop [3], as:

$$\bar{P}_{vc} \approx \frac{P_{lv}(t_{TVO}) + P_{lv}(t_{TVC})}{2} \approx 11.5 \text{ mmHg}$$

where P_{lv} is left ventricular pressure and t_{TVO} and t_{TVC} denote opening and closing times of the tricuspid valve.

First, if pulmonary vein pressure is assumed constant at $\bar{P}_{pu}$, it is clear that, during atrial filling, atrial pressure will converge to this value of $\bar{P}_{pu}$. The maximum value of atrial pressure during atrial filling (*i.e.* the peak of the v wave) can be considered as an estimation of $\bar{P}_{pu}$. This yields, from data published by Gare *et al.* [1]:

$$\bar{P}_{pu} \approx 14.3 \text{ mmHg}$$

Second, by integrating Equation (18), one can see that model resistances R_{prox} and R_{sys} can be computed as:

$$R_l = \frac{\int_T (P_{up}(t) - P_{down}(t)) \, \mathrm{d}t}{\int_T Q_l(t) \, \mathrm{d}t} = \frac{(\bar{P}_{up} - \bar{P}_{down}) \cdot T}{SV}$$

where P_{up} and P_{down} respectively denote pressures up and downstream the resistance. The integral is computed during one cardiac period T and the bar denotes the mean on one cardiac period. Using the previously mentioned reference data, this gives:

$$R_{prox} = \frac{(\bar{P}_{pu} - \bar{P}_{la}) \cdot T}{SV} \approx 0.1079 \text{ mmHg} \cdot \text{s/ml}$$
$$R_{sys} = \frac{(\bar{P}_{ao} - \bar{P}_{vc}) \cdot T}{SV} \approx 3.6144 \text{ mmHg} \cdot \text{s/ml}. \tag{S.1}$$

In case there is a valve in series with the resistance, the integral is only computed for positive values of the integrand. This allows to directly compute R_{mt} and R_{av} as:

$$R_{mt} = \frac{\int_{t_{MVO}}^{t_{MVC}} (P_{la}(t) - P_{lv}(t)) \, \mathrm{d}t}{SV} \approx 0.0278 \text{ mmHg} \cdot \text{s/ml} \tag{S.2}$$
$$R_{av} = \frac{\int_{t_{AVO}}^{t_{AVC}} (P_{lv}(t) - P_{ao}(t)) \, \mathrm{d}t}{SV} \approx 0.0849 \text{ mmHg} \cdot \text{s/ml}$$

where t_{MVO}, t_{MVC}, t_{AVO} and t_{AVC} denote opening and closing times of the mitral and aortic valves.

Finally, during diastole, Equation (21) applied to the aorta reduces to:

$$\frac{\mathrm{d}V_{ao}(t)}{\mathrm{d}t} = -Q_{sys}(t) = -\frac{P_{ao}(t) - \bar{P}_{vc}}{R_{sys}}.$$

In the previous equation, vena cava pressure has been assumed to be constant. Since $P_{ao}(t) = E_{ao} \cdot V_{ao}(t)$, this equation can be integrated to find a closed-form expression for $P_{ao}(t)$ during diastole:

$$P_{ao}(t) = \bar{P}_{vc} + (P_{ao}(t_{AVC}) - \bar{P}_{vc}) \cdot \exp\left(-\frac{E_{ao} \cdot t}{R_{sys}}\right)$$

This equation, evaluated at $t = t_{AVO}$, can be solved to compute E_{ao}.

Since not so many data was available for the right side of the cardiovascular system, we computed pulmonary artery elastance using the following approximation:

$$E_{pa} = \frac{\Delta P_{pa}}{\Delta V_{pa}} \approx \frac{PP_{pa}}{SV}$$

The first equality is the definition of elastance, *i.e.* the pressure variation caused by a volume variation divided by this volume variation. In the second equality, the pressure variation has been taken as pulmonary artery pulse pressure PP_{pa} and the corresponding volume variation has been estimated equal to the stroke volume. (This is an overestimation, as only a fraction of the stroke volume is causing an increase of arterial pressure, while the rest goes directly into the pulmonary circulation.) Pulmonary artery pulse pressure has been estimated from right ventricular pressure as:

$$PP_{pa} \approx P_{rv}(t_{PVC}) - P_{rv}(t_{PVO})$$

where t_{PVC} and t_{PVO} respectively denote closing and opening times of the pulmonary valve. We thus assume that maximum and minimum pulmonary artery pressure occur at closing and opening of the pulmonary valve, respectively.

Equation (10) describes the passive force generated by the stretching of a sarcomere unit as

$$F_{p,i}(t) = -K_i \cdot \left(1 - \frac{L_i(t)}{L_{0,i}}\right)$$

Using equations (12) and (14) linking force to pressure and sarcomere length to chamber radius, this passive force-length relationship can be converted to a passive pressure-radius relationship as:

$$\frac{P_{p,i} \cdot R_i}{2 \cdot t_i} = -K_i \cdot \left(1 - \frac{R_i}{R_{0,i}}\right) \Leftrightarrow P_{p,i} = -2 \cdot t_i \cdot K_i \cdot \left(\frac{1}{R_i} - \frac{1}{R_{0,i}}\right)$$

where $P_{p,i}$ is passive pressure. Finally, using the equation $V = 2\pi R^3/3$ to convert chamber radius to chamber volume, we obtain the following passive pressure-volume relationship:

$$P_{p,i} = -2 \cdot t_i \cdot K_i \cdot \left(\sqrt[3]{\frac{2 \cdot \pi}{3 \cdot V_i}} - \frac{1}{R_{0,i}}\right).$$

This relationship is equivalent to the notion of end-diastolic pressure-volume relationship. Using reference pressure-volume loops for the left ventricle and atrium, we could directly compute values of $R_{0,i}$ and the product $t_i \cdot K_i$ for both the left ventricle and atrium:

$$\begin{aligned} R_{0,la} &\approx 1.6611 \text{ cm} \\ R_{0,lv} &\approx 1.62 \text{ cm} \\ t_{la} \cdot K_{la} &\approx 396.012 \text{ mN/mm} \\ t_{lv} \cdot K_{lv} &\approx 30.843 \text{ mN/mm.} \end{aligned}$$

Parameter s representing the shift between maximal left atrial and left ventricular calcium concentrations was inferred from the shift between maximal left atrial and left ventricular pressures.

Parameters of the right ventricle time-varying elastance were compressed and shifted from Chung *et al.* [4] to account for a heart period of 0.45 s and to synchronize beats of the left and right ventricles.

An important but often neglected parameter of cardiovascular system models is the total stressed blood volume (SBV) in the model. More explanations on this concept can be found in [5]. In the same article, the authors state that for a human, total SBV represents 750/5500 of total blood volume. The dogs used in the experiments from which reference data was taken weighed 25 kg on average. According to [6], a 25-kg dog has a total blood volume of $25 \times 80 = 2000$ ml. If we assume that the ratio of SBV to total blood volume is the same as for a human, SBV for a 25-kg dog can be assumed to be $2000 \times 750/5500 = 273$ ml.

Step 2: iterative parameter adjustment for the systemic submodel

To simplify the parameter estimation process, the cardiovascular system model we used was split into systemic and pulmonary circulations, by assuming constant systemic and pulmonary venous pressures at $\bar{P}_{vc}$ and $\bar{P}_{pu}$, respectively. The vena cava and pulmonary veins compartments thus become points with constant pressures, implying that the two remaining subsystems, one composed of the left atrium, left ventricle and aorta and the second composed of the right ventricle and pulmonary artery, become independent. The two subsystems still have to share the same stroke volume.

The remaining four parameters of the systemic submodel, namely the parameters describing active chamber contraction (A_i) and the slopes of the passive force-length relationships (K_i) have been iteratively adjusted to match reference data coming from the previously mentioned studies. For further details, the reference data is displayed in Table S.1.

Table S. 1. Reference values for estimation of the parameters A_i and K_i.

Measurement	Value	Units	Source
Mean left atrial pressure	11.51	ml	[1]
Left atrial a wave pressure	11.74	mmHg	[1]
Maximum left atrial volume	10.79	ml	[1]
Minimum left atrial volume	7.00	ml	[1]
Maximum left ventricular pressure	106.79	mmHg	[1]
Mean left ventricular volume	20.76	ml	[2]
Stroke volume	11.67	ml	[2]

Step 3: iterative parameter adjustment for the pulmonary submodel

Because less data was available for the pulmonary submodel we could not directly compute all model parameters from reference data, as done in step 1. This was only possible for pulmonary artery elastance E_{pa}. To iteratively adjust the parameters R_{tc}, R_{pul} and E_{rv} of the pulmonary submodel, we used the proportional method developed by Hann et al. [7]. In short, this method consists in using proportional relations existing between model parameters and available measurements.

First, and to exemplify the method, for sufficiently large values of the tricuspid valve resistance R_{tc}, the following inversely proportional relation exists between R_{tc} and stroke volume SV:

$$R_{tc} \propto \frac{1}{SV}.$$

(This is related to Equation S.2 applied to tricuspid valve resistance.) Consequently, R_{tc} can be iteratively updated as

$$R_{tc,n+1} = R_{tc,n} \frac{SV(R_{tc,n})}{SV_{ref}}$$

where R_{tc}^n denotes the previous value of R_{tc}, $SV(R_{tc,n})$ is the simulated stroke volume with R_{tc} being set at $R_{tc,n}$ and SV_{ref} is the reference value of stroke volume (given in Table S.1).

Second, the following proportionality relation has been proposed [7] to iteratively estimate the value of the pulmonary resistance R_{pul}:

$$R_{pul} \propto \bar{P}_{pa}.$$

(This can be seen to be valid from Equation S.1 applied to pulmonary resistance.) As this mean pulmonary artery pressure $\bar{P}_{pa}$ was not available in the reference data we used, we based ourselves on the following approximation:

$$\bar{P}_{pa} \approx \frac{2}{3} P_{pa}(t_{PVO}) + \frac{1}{3} P_{pa}(t_{PVC}).$$

This approximation is generally used to compute mean aortic pressure [8], but we assumed it to be also true for mean pulmonary artery pressure. Then, as stated before, we could estimate $P_{pa}(t_{PVO})$ as

$$P_{pa}(t_{PVO}) \approx P_{rv}(t_{PVO})$$

since a heart valve opens when the downstream pressure equals the upstream pressure. Consequently, the relation that was used to adjust R_{pul} is

$$R_{pul} \propto P_{rv}(t_{PVO}).$$

Third, the model considers that tricuspid valve opens and closes when left ventricular pressure equals (fixed) vena cava pressure, thus we have:

$$\bar{P}_{vc} = P_{rv}(t_{TVO}) = P_{rv}(t_{TVC}).$$

Using Equation 16, this gives:

$$\bar{P}_{vc} = e_{rv}(t_{TVO}) \cdot E_{rv} \cdot V_{rv}(t_{TVO}) = e_{rv}(t_{TVC}) \cdot E_{rv} \cdot V_{rv}(t_{TVC}).$$

From the previous equation, it can be seen that end-systolic and end-diastolic ventricular volumes, respectively $V_{rv}(t_{TVO})$ and $V_{rv}(t_{TVC})$ are both inversely proportional to E_{rv}. This suggests the following proportionality relation:

$$E_{rv} \propto \bar{V}_{rv}^{-1}.$$

The reference value of mean ventricular volume $\bar{V}_{rv}$ that was used is given in Table S.2.

Table S. 2. Reference values for estimation of the parameters R_{tc}, R_{pul} and E_{rv}.

**Measurement**	**Value**	**Units**	**Source**
Stroke volume	8.93	ml	Result from step 2
Right ventricular pressure at opening of the pulmonary valve	55.33	mmHg	[3]
Mean right ventricular volume	35.35	ml	[3]

Parameter R_{pv} was kept constant at a reference value because information needed to estimate it (the derivative of the pulmonary artery waveform [7]) was not available.

Step 4: iterative parameter adjustment for venous elastances

To close the loop, the systemic and pulmonary submodels are put back together, and the venous elastances E_{vc} and E_{pu} are iteratively adjusted so that mean vena cava pressure and mean pulmonary vein pressure match the (previously constant) values computed at step 1.

Result of the parameter estimation process

Table S.3 shows that, after parameter adjustment, left atrial pressure and volume simulated by the model are in good agreement with reference values used for parameter adjustment. The same observation can be done comparing simulated left and right ventricular pressures and volumes. Please note that simulated values cannot be expected to exactly match experimental values, in particular because the reference experimental values come from three different studies.

Table S. 3. Simulated features of left atrial and left and right ventricular pressures and volumes.

Measurement	Value	Units
Mean left atrial pressure	11.70	mmHg
Left atrial a wave pressure	9.14	mmHg
Maximum left atrial volume	10.83	ml
Minimum left atrial volume	8.88	ml
Maximum left ventricular pressure	110.53	ml
Mean left ventricular volume	17.23	ml
Stroke volume	9.14	ml
Right ventricular pressure at opening of the pulmonary valve	55.73	mmHg
Mean right ventricular volume	39.05	ml

References

1. Gare M, Schwabe DA, Hettrick DA, Kersten JR, Warltier DC, et al. (2001) Desflurane, sevoflurane, and isoflurane affect left atrial active and passive mechanical properties and impair left atrial-left ventricular coupling in vivo: analysis using pressure-volume relations. Anesthesiology 95: 689–698.

2. Kass DA, Beyar R, Lankford E, Heard M, Maughan WL, et al. (1989) Influence of contractile state on curvilinearity of in situ end-systolic pressure-volume relations. Circulation 79: 167–178.

3. Maughan WL, Shoukas AA, Sagawa K, Weisfeldt ML (1979) Instantaneous pressure-volume relationship of the canine right ventricle. Circulation research 44: 309–315.

4. Chung DC (1996) Ventricular interaction in a closed-loop model of the canine circulation. Master's thesis, Rice University, Houston, Texas.

5. Burkhoff D, Tyberg JV (1993) Why does pulmonary venous pressure rise after onset of lv dysfunction: a theoretical analysis. American Journal of Physiology-Heart and Circulatory Physiology 265: H1819–H1828.

6. Finsterer U, Prucksunand P, Brechtelsbauer H (1973) Critical evaluation of methods for determination of blood volume in the dog. Pflügers Archiv 341: 63–72.

7. Hann C, Chase J, Desaive T, Froissart C, Revie J, et al. (2010) Unique parameter identification for cardiac diagnosis in critical care using minimal data sets. Computer Methods & Programs in Biomedicine .

8. Klabunde R (2011) Cardiovascular physiology concepts. Lippincott Williams & Wilkins.
